# Regulation of Macrophage Polarization by RON Receptor Tyrosine Kinase Signaling

**DOI:** 10.3389/fimmu.2014.00546

**Published:** 2014-10-31

**Authors:** Amitabha Chaudhuri

**Affiliations:** ^1^MedGenome Inc., San Francisco, CA, USA

**Keywords:** macrophages, polarization, RON signaling, tumor promotion, immune therapy

## Abstract

The M1 and M2 states of macrophage polarization are the two extremes of a physiologic/phenotypic continuum that is dynamically influenced by environmental signals. The M1/M2 paradigm is an excellent framework to understand and appreciate some of the diverse functions that macrophages perform. Molecular analysis of mouse and human macrophages indicated that they gain M1 and M2-related functions after encountering specific ligands in the tissue environment. In this perspective, I discuss the function of recepteur d’origine nantais (RON) receptor tyrosine kinase in regulating the M2-like state of macrophage activation Besides decreasing pro-inflammatory cytokine production in response to toll-like receptor-4 activation, macrophage-stimulating protein strongly suppresses nitric oxide synthase and at the same time upregulates arginase, which is the rate limiting enzyme in the ornithine biosynthesis pathway. Interestingly, RON signaling preserved some of the characteristics of the M1 state, while still promoting the hallmarks of M2 polarization. Therefore, therapeutic modulation of RON activity can shift the activation state of macrophages between acute and chronic inflammatory states.

## Introduction

Macrophages perform the essential function of preserving tissue homeostasis following infection or tissue damage in all animals in the absence of B and T cells. Macrophages originate from bone marrow-derived monocytic cells through a process of differentiation, directed by the activation of specific transcription factors (1–3). Circulating monocytes are recruited to tissues, where they differentiate into functionally distinct subsets of cells with distinct phenotypic characteristics. In response to the tissue microenvironment, these cells can either produce pro-inflammatory cytokines to kill the offending foreign pathogen and polarize T-cells to mount an adaptive immune response, or participate in tissue repair by increasing their phagocytic activity and producing growth factors for tissue healing and regeneration. The cellular plasticity is a hall mark of macrophages and the complex signaling pathways that contribute to these biochemical and functional differentiation is beginning to be understood (4, 5).

The two states of macrophage activation, “Classical” (M1) and “Alternative” (M2) occupy two extremes of a phenotypic continuum in which macrophages respond to secreted factors to evoke distinct functional responses (6, 7). These functional responses are regulated by a combination of signaling pathway modulators and transcription factors. As an example, combination of IFN-γ with toll-like receptor (TLR) pathway activation produces a complete M1 phenotype in macrophages mediated by the activation of STAT1 and NF-κB transcription factors. By contrast, IFN-γ alone causes a partial M1 response mediated by STAT1 transcription factor at sites of infection. Similarly, macrophage M2 phenotype and response are fine-tuned at tissue-specific sites by the activation of distinct sets of chemicals, such as IL-10 in the gut, or IL-4 and fatty acids in the adipose tissues (8, 9).

The activation of macrophages (AAM) into M1- or M2-type is dictated by the cytokine milieu of the tissue microenvironment. Monocytes are primed to differentiate in response to macrophage colony stimulating factor (M-CSF) or by granulocyte macrophage colony stimulating factor (GM-CSF) (2, 3). Further priming is dictated by a balance between IFN-γ and IL-4, the former pushing the macrophages into an M1 state and the latter into an M2 state. The primed macrophages receive additional signals in the form of TLR stimulation to display the full complement of classical and alternative activation functions. Whereas, M1-primed macrophages produce pro-inflammatory cytokines and inducible nitric oxide synthase (iNOS) upon TLR activation, M2-primed macrophages produce arginase, IL-10, and growth factors such as transforming growth factor-β (TGF-β), vascular endothelial growth factor (VEGF), and epidermal growth factor (EGF) among others. Interestingly, these macrophage phenotypes can be reversed and brought back to the state of naïve macrophages by growing them in the absence of any priming factors for a couple of days, indicating reversibility of the response (10, 11).

## Invoking the M1 Phenotype of Macrophages

M1 polarization can be evoked by treating naïve macrophages with a combination of IFN-γ and LPS. Macrophages express a variety of microbial pattern recognition receptors (PRRs), such as TLRs that recognize pathogen – or danger associated molecular patterns to clear the offending signal (12, 13). A large number of inflammatory cytokines including TNF-α, IFN-γ IL-12p40, IL-6 are produced rapidly after TLR-4 activation. The delayed TLR-4 response is triggered by the recruitment of TIR-containing adaptor protein (TRIF) and TRIF-related adaptor molecule (TRAM) to induce IFN-β and trigger the interferon response (14). Together, the NF-κB, p38 MAPK, and IFN-β pathways regulate the output of the TLR-4 signaling. A key enzyme of arginine metabolism, iNOS metabolizes arginine to make nitric oxide (NO), a potent anti-microbial agent. Production of NO is a hallmark of M1 macrophages and IFN-γ regulates NO production via transcriptional upregulation of iNOS (4, 15).

Built within these signaling circuits are negative feedback loops that circumscribe the intensity and duration of the LPS response. Proteins, such as dual specificity phosphatases (DUSP) and suppressor of cytokine signaling (SOCS) are induced to dephosphorylate upstream activators of the MAPK pathway and inhibit signaling downstream of the interferon receptor, respectively (6, 16). As part of body’s defense mechanism, M1 polarization is critical to mount an effective innate immune response against the offending pathogen. However, the body can sustain extensive tissue damage if the pro-inflammatory responses are allowed to persist. Therefore, these multiple feedback loops are turned on downstream of TLR-4 and cytokine-signaling pathways that quickly reduce the output from these pro-inflammatory signaling circuits (17, 18).

## Invoking the M2 Phenotype of Macrophages

Tissue resident macrophages assume an M2 phenotype by default. This phenotype, also defined as alternative AAM or M2-type of macrophages can be induced by IL-4 + IL-13 and by other signaling molecules (6, 9). In this state, macrophages metabolize arginine into ornithine by the expression of arginase-1 that diverts arginine from the production of NO and citrulline. (15). The M2 macrophages also produce growth factors and extracellular matrix remodeling enzymes that promote processes related to tissue repair and healing. Additionally, their phagocytic activity is increased to help in clearing tissue debris. The M2 activity is sustained by factors produced by injured tissues such as TGF-β and adenosine (4, 19).

## Signaling Pathways Mediating Macrophage Polarization

The signaling circuitry leading to changes in gene expression pattern during macrophage polarization is complex (16). Different subsets of tissue-specific macrophages are dependent on different signaling pathways to polarize and sustain their polarized state. As an example, c-jun N-terminal kinase pathway (JNK) is required for the adipose tissue-associated macrophages to assume M1-phenotype (20). Polarization of macrophages by the phosphatidyl inositol-3 kinase (PI3K) pathway is mediated by the activation of AKT1 and AKT2 kinases. Genetic ablation experiments revealed that AKT1 and 2 regulate macrophage M1 and M2 phenotype in a reciprocal pattern (21, 22). In the absence of AKT1, macrophages produce pro-inflammatory cytokines resembling the M1 phenotype, whereas in the absence of AKT2 the cells express markers of M2 polarization such as Arg-1, Fizz-1, and IL-10 (21, 23, 24). Interestingly, preliminary data support that this reciprocal regulation of macrophage M1/M2 phenotypes is mediated by a micro-RNA, mir-155, and its target transcriptional regulator CAAT-enhanced binding protein-β play an important role (23). JAK/STAT pathway downstream to IFN-γ is a strong inducer of M1 polarization, although to reach the full spectrum of the M1-phenotypic state, dual activation of the TLR-4 and IFN-γ pathways are required (25, 26).

Signals that promote M2 polarization are diverse and a variety of molecules from cytokines to growth factors can influence this transition (6). The IL-4/IL-13 combination is a physiological mediator of the M2 state that impinges on the transcription factor STAT-6 to induce cell surface expression of M2 markers and metabolic reprograming (9). In the absence of IL-4/IL-13, M-CSF and IL-10 can push macrophages to assume an M2 phenotype mediated by the transcription factor STAT-3 and SP-1, respectively. In this state, the macrophages become highly phagocytic, produce growth factors that promote repair of wound or tissue damage, and promote Th-2 immune response. Fcγ receptors in combination with LPS promotes Th-2 response, upregulates antigen presentation, turns off IL-12, and induces IL-10 production by activating the Syk and PI3K pathways that cross-talk with TLR signaling. Finally, glucocorticoids promote macrophage adherence, spreading, phagocytosis, induction of complement proteins, and secretion of IL-10 by directly engaging the macrophage transcription machinery.

Integrating the function of M1 and M2 macrophages in a physiological setting raises several questions:

1. Are the M1 and M2 macrophage states mutually exclusive in a tissue environment?
2. Can a cell transition from one state to the other directly, or are there other intermediate states?
3. Can a macrophage assume characteristics of both M1 and M2 states? How do they arise? Do they represent a fleeting intermediate, or can cells in this state be stabilized?

Answers to these questions are not fully known. However, M1- and M2-polarized macrophages are found as mixed populations in the tissue microenvironment. Depending on the inflammatory stimuli, one state may dominate over the other. Both M1 and M2 states are functionally and phenotypically heterogeneous. The translatability of macrophage M1/M2 polarization in normal homeostasis and in diseases have remained elusive beyond the fact that M1 macrophages favor bacterial and viral elimination, whereas M2 macrophages give protection against helminthes and other parasites and participate in tissue repair. Observations *in vitro* cannot always be readily applied to *in vivo* situations and many of the macrophage responses discussed above are yet to find validation *in vivo* (27).

## How Functional States of Macrophages are Modulated by Growth Factor Signaling

So far in our discussion, the macrophage phenotypic and functional states have been described in the context of inflammation or interaction between pathogens and immune cells. However, M2-polarized macrophages also perform essential functions in the resolution of tissue inflammation, remodeling of the tissue microenvironment during wound healing and repair of tissue damage. In the next section, the effect of growth factor signaling on macrophage polarization, and its implication in human cancer is discussed.

**Figure 1:**
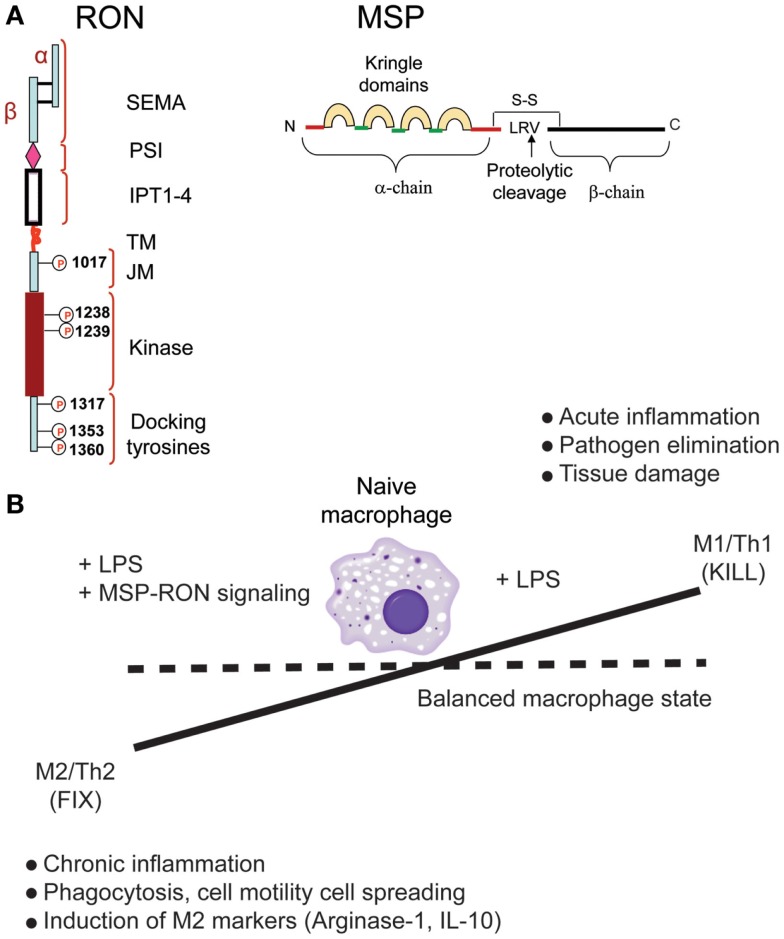
**Recepteur d’origine nantais (RON) promotes macrophage M2 polarization**. **(A)** Structure of RON and MSP proteins. **(B)** RON signaling alters macrophage phenotype and restores the balance between M1- and M2-polarized states.

Among the growth factor receptors known to modulate macrophage behavior and function is the receptor tyrosine kinase “recepteur d’origine nantais” (RON) (28, 29). The ligand for RON, macrophage-stimulating protein (MSP) regulates macrophage motility and its phagocytic activity (28). MSP is produced by the liver and circulates in an inactive form in the serum. MSP activation occurs as a result of proteolytic processing of Pro-MSP resulting in active MSP. Several trypsin like proteases such as matriptase, hepsin, and hepatocyte growth factor-A (HGF-A) cleave inactive pro-MSP into an active form (30) (Figure 1A). These proteases are known to be activated at sites of inflammation and can be a source of active MSP that can turn on RON signaling on macrophages and epithelial cells at these sites.

Genetic ablation of RON kinase activity in mice leads to viable and fertile progenies with no apparent developmental defects. However, studies showing that RON knockout (RON-KO) mice are sensitive to LPS challenge suggested that RON signaling negatively regulates downstream effects of TLR-4 activation (31, 32). Further studies by many groups have led to a general model in which RON signaling promotes some of the functional and phenotypic traits of M2-like macrophages, in particular, a strong suppression of pro-inflammatory cytokine production in response to LPS (33, 34) or LPS + IFN-γ (35), suppression of iNOS (36), induction of arginase-1 (37, 38), and expression of scavenger receptors (34). Interestingly, treatment of peritoneal macrophages with MSP alone induced activation of MAPK and PI3K pathways, but failed to induce any of the hall marks of M2 polarization such as expression of arginase-1, scavenger receptors, or IL-10 (34). When MSP was combined with LPS stimulation, macrophages exhibited hallmarks of M2-polarized state (Figure 1B). Further, global gene expression analysis and measurement of cytokines in the conditioned media indicated that RON signaling had minimal effect on TLR-4-mediated early NF-κB activation – the effect being significantly pronounced at later time points (34).

Taken together, these observations support that RON signaling has dual effect on macrophages. On the one hand, it enhances macrophage motility and survival without any input from TLR signaling, but on the other hand, it significantly modifies the TLR-4 signaling output when LPS is present along with MSP. Interestingly, the reprograming of the TLR-4 signaling circuits by RON is sensitive to mice genetic background (34).

## Impact of Genetic Background and Differential Effect of RON on Macrophage Polarization

Studies employing different mice strains have revealed that host genetic background significantly influences the metabolic reprograming and behavior of macrophages when exposed to LPS or IFN-γ (39). Mills et al. first reported that whereas macrophages from C57Bl/6 mice produced citrulline and nitric oxide (NO) in response to LPS or IFN-γ, those from BALB/c background produced ornithine (40). Both NO and ornithine are the products of differential arginine metabolism mediated by the expression of enzymes iNOS and arginase-1, respectively, and this “fork in the arginine metabolism” is recognized as one of the hallmarks of M1 and M2 polarization (4, 39). Gene expression profiles comparing bone marrow-derived macrophages from different mice strains further revealed that in response to LPS, timing and intensity of expression of genes differed significantly between mice strains (41).

**Figure 2:**
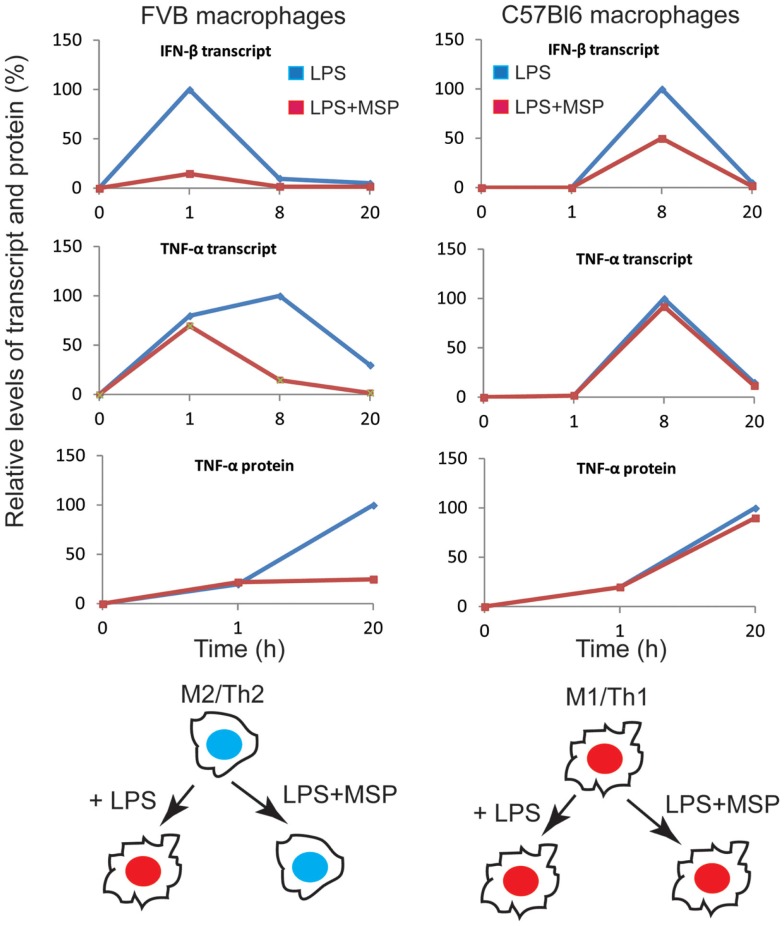
**Overview of the impact of the RON pathway on M1 versus M2 differentiation program in the context of TLR-4 signaling**. Transcript and protein levels of IFN-β and TNF-α were compiled from experimental values. Protein or mRNA levels at each time point are expressed as percentage of maximal expression (100%). We propose that RON signaling in macrophages from FVB mice preserves M2 differentiation in the presence of TLR-4 signaling, whereas C57Bl6 macrophages maintain polarization toward M1 cells in the presence of RON signaling. Taken from Chaudhuri et al. (34).

Interestingly, RON signaling modulated the TLR-4 responses of macrophages between C57Bl/6 (M1-polarized) and FVB (M2-polarized) mice differently (34). Whereas MSP strongly suppressed LPS-induced production of pro-inflammatory cytokines in macrophages from FVB background, the effect was minimal in the C57Bl/6 background. A clue to the mechanism came from analyzing the effect of RON signaling on LPS-induced gene expression in macrophages. LPS induced the transcriptional targets of the NF-κB and MAPK pathways early on, and RON signaling had no effect on most of the early response genes. The late response genes, dominated by the transcriptional targets of interferon signaling, selected NF-κB target genes, and genes associated with tissue repair and immune tolerance was modulated variably by RON. RON suppressed most of the targets of the interferon pathway (50% of the downregulated genes at the later time point were targets of interferon signaling), as well as few selected targets of the NF-κB pathway (TNF-α), but enhanced the expression of tissue repair (EGF, PDGF, MMP9) and immune tolerance genes (IL-10, IL-19, CTLA-2A). The kinetics of IFN-β expression in response to TLR-4 activation was rapid (1 h) in macrophages from FVB mice, whereas it was significantly delayed (8 h) in C57Bl/6 background. This early upregulation of IFN-β in FVB mice was blunted by RON signaling resulting in a strong inhibition of the expression of interferon regulatory factors (IRFs) and target genes of the interferon pathway at later time points. The IFN-β expression however, was minimally affected by RON signaling in C57Bl/6 macrophages partly because of the delayed expression of IFN-β in this strain background, and partly as a result of high expression of TNF-α, IL-12, and IL-6, which by themselves can modulate the expression of IFN-β independently of LPS (34). The final outcome of the interplay between RON and LPS signaling in these two mice strains resulted in the stabilization of M2-polarized state in FVB mice even in the presence of a strong M1-polarizing signal, but failed to alter the phenotype of macrophages from C57Bl/6 background (Figure 2).

In addition to affecting the polarized behavior of macrophages, RON kinase-deficient FVB mice formed less number of tumors, which developed with a delayed kinetics in two models of chemical induced carcinogenesis (34, 42). This inhibitory effect was lost in the C57Bl/6 RON-KO background (34). Additionally, depletion of CD8^+^ T-cells in a transplantable fibrosarcoma model in FVB mice suppressed the rejection of tumors in the RON-KO background suggesting that lack of RON in the innate immune compartment facilitates generation of a CTL response against the tumor (34). These observations support the hypothesis that ablation of RON function in the innate immune compartment accentuates tumor-specific T-cell responses.

In the last few years, tumor-associated macrophages have received significant attention due to their pro-tumorigenic properties, such as producing tumor-promoting and pro-angiogenic factors and suppression of the adaptive immune response within the tumor microenvironment (43). Polarization of macrophages into an M2-type is one of the mechanisms that subverts the sentinel function of the innate immune cells and make them pro-tumorigenic.

How can this immune-modulatory property of RON be reconciled with normal tissue homeostasis? Maintaining macrophages in M1/M2-like polarized state is important under certain physiological conditions. For example, during wound healing or during repair of damaged tissues, macrophages serve two important functions. First, it is ready to mount an immune response to eliminate pathogens, if the wound site gets infected, and during the same time limit the intensity and duration of the localized immune response to prevent further tissue damage. Second, it needs to produce growth-promoting and tissue-rebuilding factors to accelerate healing and reverse damage. In this context, the tissue repair or wound healing pathways of macrophages are co-opted by the tumor to promote its own survival against immune attack and activation of RON in the tumor microenvironment may facilitate this conversion.

## Conclusion and Therapeutic Implications

M1 and M2 polarization of macrophages is dynamically controlled by changes in the tissue microenvironment. These two functional states participate in two important activities – protection against foreign pathogens and promotion of tissue restoration and healing after injury. Therefore, tight regulation of these two states is critical to the health of the organism (Figure 1B). Sustained activation of M1 state can lead to excessive tissue damage as a result of excessive inflammation, whereas prolonged activation of the M2 state can cause chronic inflammation leading to cancer. Therapeutic targeting of certain diseases may involve artificial manipulation of macrophage polarization. As an example, inhibiting RON function in tumor-associated macrophages can restore tumor immunity allowing enhanced efficacy of cancer immunotherapy drugs. Similarly, enhancing RON activity in tissue-associated macrophages can lead to efficient wound healing and restoration of tissue damage. However, such artificial manipulation of immune cell functions has to be tightly controlled to prevent systemic damage to the organism.

## Conflict of Interest Statement

The author declares that the research was conducted in the absence of any commercial or financial relationships that could be construed as a potential conflict of interest.
